# An Account of a Particular Change of Structure in the Human Ovarium

**Published:** 1789

**Authors:** Matthew Baillie


VIII. *An Account of a particular Change of Structure in the human Ovarium. By* Matthew Baillie, *M. D.*—From the *Philosophical Transactions of the Royal Society of London*, Vol. LXXIX. for the Year 1789. Part I. 4to. London, 1789.

THE ovaria in women are ſubject to a great variety of changes from their natural ſtructure. Many of theſe are exactly ſimilar to what take place in other parts of the body; but there is one which ſeems peculiar to them, the nature of which has probably not been hitherto very well aſcertained. The change of ſtructure to which I allude is a converſion of the natural ſubſtance of an ovarium into a fatty maſs, intermixed with hair and teeth. This ſort of change is rare, although it occurs ſufficiently often to have been ſeen by moſt perſons who are very converſant in the examination of dead bodies. There are many caſes of it related in the different books of diſſections, but, as far as I have diſcovered, moſt commonly without any remarks upon the mode of formation*; or they

* It has been the opinion of ſome, that hair, teeth, nails, feathers, &c. are animal vegetables or plants; and, agreeably to they have been confidered as very imperfect attempts at the growth of a fœtus in the ovarium, in confequence of connexion between a male and a female. This conjecture refts no doubt on ftrong circumftances of probability, and yet there are many powerful reafons which feem to oppofe its being well founded. Generation is a procefs always depending on the action of a certain caufe, viz. the ufual connexion between a male and a female; and, when effects fimilar to thofe in generation are perceived, it becomes very natural to conclude that this caufe has been employed. The bias to fuch an opinion will become the ftronger, from reflecting on the paffions that are known to influence fo powerfully mankind, by which the agency of this caufe is frequently excited. When a change, therefore, was obferved in an ovarium, by which it was converted into a fatty mafs, with hair and teeth, this fhould feem to correfpond fo much with a change taking place in confequence of generation, that the mind would fcarcely enterto this opinion, Dr. Tyfon confiders the growth of hair and teeth in the ovarium as a *lufus naturæ*, where nature endeavours to produce fomething, and, being difappointed in forming an animal, produces a vegetable.—Vide Hooke's Lectures and Collection, No. II. p. 11 and 15.

tain a doubt of its arising from the same cause, and would readily infer that it had been preceded by a connexion between the sexes. This doubt would still be less, from the circumstance of a complete fœtus being sometimes formed in the ovarium where the usual means of generation had been employed. The following case, however, exhibits many reasons why we should be led to believe that the ovaria in women have some power within themselves of taking on a process which is imitative of generation, without any previous connexion with a male; and it is with this view that I proceed to relate it.

In a female child, about twelve or thirteen years old, which was lately brought to Windmill Street for dissection, I found the right ovarium converted into a substance, doughy to the touch, and about the size of a large hen's egg. Upon cutting into the substance, I found an apparently fatty mass, intermixed with hair and an excrescence of bones. This startled me very much, as I had always been led to believe that such appearances were a sort of imperfect conception. The circumstances altogether being very singular, I was led to pay considerable attention to the change in the ovarium.

The fatty mafs was of a yellowifh white colour, in fome places more yellow than in others, was very unctuous to the feeling, and confifted of fhortened or feparated particles, not having the fame coalefcence which the fat has generally in the body. It became very foft when expofed to the heat of a fire, and funk into a portion of paper, on which it was fpread, fo as to make it more tranfparent. When the paper to which it was applied was expofed to the flame of a candle, it burnt with confiderable crackling.

The hair with which the fatty fubftance was mixed grew out of the inner furface of the capfule containing it, in fome places in folitary hairs, but chiefly in fmall fafciculi, at fcattered irregular diftances. Befides thefe, there were loofe hairs involved in the fatty mafs. The hairs were, fome of them, of confiderable length, even to three inches, were fine, and of a light brown colour. They refembled much more the hairs of the head than what are commonly found on the pubis, and correfponded very much in colour to the hair of the girl's head.

There arofe alfo from the inner furface of the capfule fome veftiges of human teeth. One appeared to be a canine tooth, another to be a fmall

ſmall grinder, two others to be inciſors, and there was alſo a very imperfect attempt at the formation of another tooth. Theſe were not fully formed, the fangs being wanting; but in two of them the bodies were as complete as they are ever found in the common circumſtances. They were each of them incloſed in a proper capſule, which aroſe from the inner ſurface of the ovarium, and conſiſted of a white thick opaque membrane. Attached to the capſules of three of the teeth there was a white ſpungy ſubſtance. The membrane of the ovarium itſelf was of ſome conſiderable thickneſs, but unequal in the different parts, was very ſmooth in its inner ſurface, and more irregular externally. The uterus was ſmaller than it is commonly at birth, was perfectly healthy in its ſtructure, and upon opening into its cavity it exhibited the ordinary appearances of a child's uterus at that period. The left ovarium was very ſmall, correſponding to the ſtate of the uterus. It appears clearly from this that the uterus had not yet received the increaſe of bulk which is uſual at the age of puberty. The hymen was entire, ſuch as is commonly found in a child of the ſame age; and there was juſt beginning a *lanugo* upon the labia, not more than what is often found on the upper lip of a boy of fifteen years old. Such are the circumſtances attending this ſingular caſe, and they preſent to the mind various grounds of conſideration.

The formation of hair and teeth is a ſpecies of generation, for in fact it makes a part of it, and ſtrikes the mind as being very different from any irregular ſubſtance which is formed by diſeaſe. This formation, too, takes place in a part of the body which is ſubſervient to generation, and where a complete fœtus is ſometimes formed. The whole of this looks very much as if the production of hair and teeth in the ovarium was a ſort of imperfect impregnation. But when we take another view of it, there are reaſons at leaſt equally ſtrong for believing that ſuch productions may ariſe from an action in the ovarium itſelf, without any ſtimulus from the application of the male ſemen.

In the caſe before us the uterus was as ſmall as at birth, indeed more ſo, and the left ovarium (which was perfectly healthy) correſponded to the ſtate of the uterus. It had not been at all ſtimulated, nor did appear capable of being ſtimulated, by the application of the male ſemen. This ſeems to be a ſtrong circumſtance; for in a caſe where there was an ovum formed in in one of the Fallopian tubes, the uterus was enlarged to more than twice its unimpregnated fize, and, upon opening into its cavity, the decidua was obferved to be formed as completely as in the impregnated uterus. This preparation is ftill preferved in the collection of Windmill Street. Nothing can be a ftronger proof, that when an impregnation takes place out of the cavity of the uterus, the uterus ftill takes a fhare in the action, and undergoes fome of the changes of impregnation. In another preparation, which is preferved in the fame collection, where there was a fœtus formed in the ovarium, the uterus was increafed to more than twice its ordinary fize, was very thick and fpungy, and had its blood veffels enlarged as in an impregnated uterus. This becomes another very ftrong proof of the action of the uterus in the formation of an extra-uterine fœtus. In the cafe before us, however, the uterus had undergone no change, and does not feem to have arrived at that period when it could be capable of undergoing fuch a change.

Befides we are not to confider the formation of teeth in the ovarium to be a quicker procefs than it is commonly in the head of a fœtus; but in the prefent cafe the teeth having advanced fully as far as they are at ſome months after birth, this proceſs muſt have begun at leaſt more than a twelvemonth before the death of the child. If then we conſider it as an impregnation, ſince the appearances of the child do not warrant us to believe her to have been more than twelve or thirteen years old, this brings the date of the impregnation to an earlier period than can well be believed. From all theſe circumſtances we might be led to ſuppoſe that the formation of the hair and teeth was not in conſequence of any connexion with a male, but aroſe from ſome action of the ovarium itſelf, in which the uterus did not participate. The exiſtence of the hymen, eſpecially in ſo young a girl, becomes a collateral confirmation of the ſame opinion, although much is not to be reſted on it when taken ſingly.

It will, perhaps, have ſome influence in removing the prejudices againſt this opinion, to make the following remarks:—Hair is occaſionally formed in parts of the human body which are abſolutely unconnected with generation. Encyſted tumours are ſometimes found containing hair. Mr. Hunter has a preparation of this ſort in his collection, which he cut out from under the ſkin of the eyebrow. This tumour was perfectly complete, and unconnected with the ſkin, except by the common intervention of cellular membrane, ſo as to have no communication whatever with the hair of the eyebrow. In this inſtance there was certainly a ſpecies of generation taking place in the encyſted tumour itſelf, forming hairs as completely and fully as in the common progreſs of the formation of a child. Such encyſted tumours have been found in other parts of the human body, and ſtill more frequently in quadrupeds. Mr. Hunter has in his collection many ſpecimens of encyſted tumours from cows and ſheep containing hair and wool. Theſe were perfectly complete, ſo as to have poſſeſſed a power of production within themſelves, and were many of them found deeply ſeated at a conſiderable diſtance from the ſkin, which is the common parent of hair. In theſe tumours there is often the appearance of layers of cuticle, which is probably a preparatory ſtep to the formation of hair. All this ſhows moſt clearly that hair may be formed without any ſpecies of generation, as it is commonly underſtood.

But hair is in itſelf as diſtinct a conſequence of generation as teeth, and as much a peculiar

ſubſtance. If, then, the one be formed, there appears to be no reaſon why the other ſhould not alſo be formed. The action producing the one is not better underſtood than that producing the other; nor does it appear to be really in itſelf leſs connected with that ſpecies of generation ariſing from the approach of a male, ſo that teeth may probably be formed by a peculiar action taking place in the ovarium itſelf as well as the hair.

It will tend to add farther weight to this opinion, to conſider that many of the adult teeth are formed in a child after birth, and therefore their formation depends on an action taking place in the jaws at a particular period, and not on original growth. The ſame circumſtance ſtrikes more ſtrongly in the occaſional formation of teeth at an advanced time of life. Both of theſe proceſſes take place after the animal has been formed, in conſequence of a certain action being excited in a particular part of the body, and therefore there is leſs difficulty in believing that the ſame ſort of proceſs may go on in another part of the body not commonly employed in it. It ſeems reaſonable alſo to ſuppoſe, that the ovaria ſhould have a greater aptitude of taking on a proceſs ſomewhat ſimilar to generation than the other indifferent parts of the body, as they conſtitute a part of the organs which are ſo materially concerned in the real proceſs itſelf*. Theſe circumſtances, when taken collectively, would ſeem to render it very probable that the formation of hair and teeth in the ovarium does not neceſſarily depend on a connexion between a male and a female, (as has been the common opinion) but ariſes from ſome action within the ovarium itſelf which is imitative of generation.

